# Amplicon Sequencing Reveals Microbiological Signatures in Spent Nuclear Fuel Storage Basins

**DOI:** 10.3389/fmicb.2018.00377

**Published:** 2018-03-09

**Authors:** Christopher E. Bagwell, Peter A. Noble, Charles E. Milliken, Dien Li, Daniel I. Kaplan

**Affiliations:** ^1^Earth Systems Science Division, Pacific Northwest National Laboratory, United States Department of Energy, Richland, WA, United States; ^2^Department of Periodontics, University of Washington, Seattle, WA, United States; ^3^Environmental Sciences and Biotechnology, Savannah River National Laboratory, United States Department of Energy, Aiken, SC, United States

**Keywords:** bacterial diversity, spent nuclear fuel, aluminum, amplicon, signatures

## Abstract

Water quality is an important determinant for the structural integrity of alloy cladded fuels and assemblies during long-term wet storage. Detailed characterization of a water filled storage basin for spent nuclear reactor fuel was performed following the formation and proliferation of an amorphous white flocculent. White precipitant was sampled throughout the storage basin for chemical and spectroscopic characterization, and environmental DNA was extracted for 454 pyrosequencing of bacterial 16S rRNA gene diversity. Accordingly, spectroscopic analyses indicated the precipitant to be primarily amorphous to crystalline aluminum (oxy) hydroxides with minor associated elemental components including Fe, Si, Ti, and U. High levels of organic carbon were co-localized with the precipitant relative to bulk dissolved organic concentrations. Bacterial densities were highly variable between sampling locations and with depth within the water filled storage basin; cell numbers ranged from 4 × 10^3^to 4 × 10^4^ cells/mL. Bacterial diversity that was physically associated with the aluminum (oxy) hydroxide complexes exceeded an estimated 4,000 OTUs/amplicon library (3% cutoff) and the majority of sequences were aligned to the families *Burkholderiaceae* (23%), *Nitrospiraceae* (23%), *Hyphomicrobiaceae* (17%), and *Comamonadaceae* (6%). We surmise that episodic changes in the physical and chemical properties of the basin contribute to the polymerization of aluminum (oxy) hydroxides, which in turn can chemisorb nutrients, carbon ligands and bacterial cells from the surrounding bulk aqueous phase. As such, these precipitants should establish favorable microhabitats for bacterial colonization and growth. Comparative analyses of 16S rRNA gene amplicon libraries across a selection of natural and engineered aquatic ecosystems were performed and microbial community and taxonomic signatures unique to the spent nuclear fuel (SNF) storage basin environment were revealed. These insights could spur the development of tractable bio-indicators that are specific of and diagnostic for water quality at discrete locations and finer scales of resolution, marking an important contribution for improved water quality and management of SNF storage facilities.

## Introduction

Over the past four decades, the nuclear power industry has produced over 76,000 metric tons of spent nuclear fuel (SNF) (National Research Council [NRC], 2014). This spent fuel is typically stored onsite at the nuclear power plant facilities in water filled storage basins and more recently, due to lack of space, some of the fuel has been transferred to dry cask storage. Approximately 78% of the SNF in the United States is held in wet storage and the remaining 22% in dry casks. A typical nuclear power plant generates annually 20 metric tons of SNF and the total annual generation of spent fuel in the United States is about 2,000–2,300 metric tons. In addition to SNF originating from the nuclear power industry, there is a much smaller (357 metric tons) and slower growing volume of SNF originating from the United States Department of Energy’s (DOE) weapons production facilities (United States Department of Energy [DOE], 1995). Understanding the long-term fate and stability of SNF assemblies in these storage facilities is becoming increasingly important.

After the production life of nuclear fuel, spent fuel rods are most commonly transferred to wet storage in water filled reinforced concrete basins. Wet storage allows the fuel to naturally decay dangerous radionuclides and to safely disperse heat. SNF may remain in wet storage for up to a decade or longer before being reprocessed or converted to long-term dry storage configurations. Nuclear fuel rods are composed of corrosion resistant cladding that prevents the release of fission products during reactor operation and storage. Aluminum alloy was the cladding material of choice for defense related production reactors in the United States and was also extensively used in research reactors. The majority of reactor configurations since have adopted austenitic stainless steel or zirconium alloys for fuel cladding. Aluminum alloys can be remarkably resistant to corrosion in aqueous environments due to the chemical formation of protective aluminum oxide films on the metal surface. The primary mechanisms by which these aluminum oxide films deteriorate are electro-chemical, chiefly ionic displacement (Cl^-^, NO_3_^-^, SO_4_^2-^, PO_4_^2-^) of the oxide barrier leading to pitting and galvanic corrosion (Pešić et al., 2004). Monitoring water-quality parameters to provide measures of early signs of assembly deterioration/corrosion are essential during long-term wet storage (International Atomic Energy Agency [IAEA], 1998).

The potential for electrochemical corrosion of SNF is obviously an important concern; however, strict focus on the management of bulk water physical–chemical factors (temperature, conductivity, Cl^-^ levels, and radioactivity) may have a tendency to ignore the fact that microbiological pathways can also affect the stability of fuel components during wet storage (e.g., Wolfram et al., 1996; Santo Domingo et al., 1998). Microorganisms have been described previously for inhabiting SNF storage facilities, as well as for colonizing aluminum surveillance coupons and even surfaces of fuel components (Pritchard, 1997; Santo Domingo et al., 1998). Microbial induced corrosion (MIC) could contribute to or exacerbate the corrosion of fuel components during wet storage (Wolfram et al., 1996); however, the actual significance of MIC of SNFs during long-term wet storage has been debated (Louthan, 1997). The metabolic activity of diverse groups of bacteria (e.g., fermenters, metal respiring anaerobes) can initiate, participate, or accelerate pitting and corrosion of aluminum cladding in wet storage. Aggressive chemical treatments are employed to reduce planktonic growth and biofilm formation in SNF storage basins holding stainless steel, zirconium alloy, or carbon steel cladding materials; however, many chemical disinfectants accelerate the corrosion of aluminum cladding and therefore are not permissible. Consequently, long-term management of storage basins containing aluminum clad fuel components require more thorough characterization and monitoring of both, microbiological and water quality parameters to ensure fuel component stability during prolonged wet storage.

About 16% or 56.3 metric tons of the DOE’s inventory of SNF is located at the Savannah River Site (SRS) in Aiken, SC, United States. The SRS spent fuel disassembly basin (described in more detail below) contains approximately 18,000 fuel assemblies; roughly 90% of which is comprised of aluminum, stainless steel, and zirconium cladded fuels. The aluminum based fuels were generated from defense (highly enriched fuel) and research (low enriched fuel) from domestic and foreign sources. The balance of the basin’s inventory is composed of high flux isotope cores that were used for research and the production of heavy element isotopes. Strict management of water chemistry continuously restricts the accumulation of reactive ions that are corrosive to aluminum alloy cladded fuels. The basin water is monitored by enrichment assays to evaluate the relative abundance of anaerobic and fermentative bacteria as a leading indicator for MIC (Santo Domingo et al., 1998); however, the limitations of growth dependent methodologies are generally well recognized (Staley and Konopka, 1985). A reliance on laboratory cultivation does not provide an accurate analysis of the status, composition, or density of the microbial community and likely misses the most problematic microbes in this system, particularly those actively attached to metal surfaces. A limited number of studies have explored microbiological diversity in SNF storage basins but a thorough investigation of microbial communities inhabiting these facilities has not been performed; thus, our knowledge of all the potential interaction pathways between diverse microbes and spent nuclear materials remains a ripe area for investigation. Microbial activity can have a strong influence on water chemistry and fuel stability, therefore monitoring these features in direct association with aluminum alloy cladded fuels is important. In this study, we employed next generation sequencing to survey the diversity and complexity of the microbial community in a SNF storage facility at a time when an anomalous white precipitant was beginning to appear throughout the basin and in association with stored nuclear materials. The goals of this study were twofold. First, we set out to determine if the appearance and maturation of the precipitant throughout the basin was an emergent phenomenon signifying biofouling or an important change in chemical conditions on a localized spatial scale that would not be readily detected by routine bulk water sampling. Secondly, computational analytics were used to test the hypothesis that the environmental forces of this built environment imprint tractable signatures on the indoor microbiome that can distinguish the SNF storage pool microbial community from other environmental datasets. This study marks a starting point for the emergence of microbiological diversity patterns (signatures) that could be reliably monitored for an improved understanding of the coupled microbiological–chemical–physical dynamics in this complex engineered environment.

## Materials and Methods

### Basin Sampling

The SNF disassembly basin at the Savannah River Site (Aiken, SC, United States) is a concrete pool that ranges in depth from 5 to 15 m and holds approximately 13,000 m^3^ of water. Water in the basin cycles between 18 and 26°C in accordance with spent fuel shipments and is continuously circulated through a deionizing system containing 3.4 m^3^ of ion exchange resin and a 1 m thick sand bed for particulate filtration. The circulation rate through the deionizing system is 0.76 m^3^ min^-1^ and 7.6 m^3^ min^-1^ through the sand filter bed; thus, the calculated turnover of the basin would occur every 12 days and 28 h, respectively. Makeup water is added to the basin as needed to replace evaporative losses, on average 45 m^3^/month. Typical water chemistry in the basin is 1.5 μS/cm conductivity, pH 6.1, chloride <0.05 mg/L.

For this study, water and precipitant samples were collected from 13 different locations and depths throughout the SNF storage basin using a peristaltic pump followed by sequential passage through an autoclaved bag filter (10 μm pore size) and a sintered metal mesh filter (1 μm pore size). Representative samples were held at 4°C and -20°C for preservation prior to analysis.

### Total Carbon Analysis

In addition to water and precipitant samples collected from the storage basin proper, water was also collected from various operational units such as the back-flush settler basin, the packed sand bed filter, and the deionizer system for carbon analysis. Water samples and gravity filtered precipitant were pretreated by mild sonication and passage through 0.45 μm syringe filters. Filtrates were processed by an OI Analytical (College Station, TX, United States) 1020A Total Carbon Analyzer for total carbon (TC) = total organic carbon (TOC) + total inorganic carbon (TIC) measurement. Calibration standards were prepared fresh using potassium hydrogen phthalate and a sodium carbonate solution.

### Inductively Coupled Plasma Atomic Emission Spectroscopy (ICP/AES)

Inductively coupled emission spectrometry (ICP/AES) was used to analyze composite retentates of the precipitant sampled throughout the basin. Aqua regia [3:1 (vol:vol) mixture of HCl and HNO_3_] and sodium peroxide fusion digestions were performed (30 min) on independent precipitant samples. Digestion reactions were diluted to 10 ml in deionized water and analyzed on an Agilent 730 ES Simultaneous Inductively Coupled Plasma–Atomic Emission Spectrometer (ICP–AES). Yttrium was used as an internal standard and instrument calibration was performed with a NIST traceable elemental standard.

### X-Ray Diffraction (XRD)

Mineralogy was determined by X-ray diffraction (XRD) analyses of the <2-μm fraction. XRD samples were subjected to three sequential heat treatments for mineral identification: 16 h at 25°C, 16 h at 65°C, and then 16 h at 300°C.

### Total Bacterial Cell Counts

Basin water samples (50 mL) were thoroughly mixed by hand shaking. Serial dilutions were spotted onto microscope well slides (3 wells/sample) and heated to dryness at 65°C. Slides were stained for 2 min with fluorescein isothiocyanate [0.04% FITC prepared in a 0.5 M NaCO_3_ – phosphate buffer (pH 7.2)], rinsed with sterile filtered deionized water and air dried at ambient room temperature. Cells were counted by epi-fluorescent microscopy at 1000× magnification.

### DNA Extraction

Precipitant was suspended in a minimal volume of basin water and homogenized by vigorous shaking. Aliquots (1 mL) were pelleted by sequential centrifugation at 10,000 × *g* for 3 min. Genomic DNA was extracted using both the Wizard^®^ Genomic DNA Purification Kit (Promega; utilizes chemical lysis) and the PowerBiofilm^®^ DNA Isolation Kit (Mo Bio; utilizes a combination of mechanical and chemical lysis) per the manufacturer’s instructions in an effort to minimize methodological bias. Multiple independent extractions (*n* = 6) were conducted per kit. DNA yields were quantified using the NanoDrop 1000 spectrophotometer (Thermo Scientific) and DNA extracts were pooled prior to sequencing analysis.

### Amplicon 454 Pyrosequencing

In an effort to minimize primer set specific biases and ensure maximum community coverage, two different hypervariable regions of the 16S rRNA gene were targeted for amplicon pyrosequencing of the SNF basin microbial community. Universal bacterial PCR primers 27-F and 338-R which span the V1–V2 regions (Fierer et al., 2008; Lauber et al., 2009) and primers 520-F and 802-R that span the V4–V5 regions (Vaz-Moreira et al., 2011) were used; both primer sets have been demonstrated for pyrosequencing. Fusion primers were synthesized for compatibility with the GS FLX Titanium Sequencing Kit according to the manufacturer’s instructions (454 Life Sciences, Roche). The thermal cycling program began with an initial denaturation at 95°C (5 min); followed by 35 cycles of denaturation at 94°C (30 s), annealing at 50°C (1 min), and extension at 72°C (1 min). A final extension step was conducted at 72°C for 10 min. PCR reactions (50 μl) used the HotStar HiFidelity Polymerase Kit (Qiagen), 1.0 μM (final concentration) of each primer, and 1.2, 12, 25, and 50 ng DNA template. All PCRs were conducted in triplicate. Amplification product specificity was assessed using the FlashGel^TM^ System (Lonza) on 1.2% agarose gels and purified by the QIAquick PCR Purification Kit (Qiagen). Purified samples were pooled and delivered to the Environmental Genomics Core Facility at the University of South Carolina (Columbia, SC, United States) for pyrosequencing on a 454 Life Sciences GS FLX System (Roche).

### Bioinformatic Analysis

Using both the fasta and quality files, the reads were trimmed to have a minimum length of 100 bp, a maximum number of homopolymers of 10 and a minimum quality score of 25 using the trim.seqs command in Mothur (Schloss et al., 2009). Chimeras were removed from the trimmed reads using the SILVA gold alignment standard and uchime (Edgar et al., 2011). The resulting reads were hand-curated using Seaview (Gouy et al., 2010), dereplicated to identify unique sequences using Splicer 1.1. The distances of the sequences were calculated using Psi-distance 1.0. The Splicer and Psi-distance were downloaded at http://tornado.igb.uiuc.edu. The distances were clustered using unweighted-pair group method using average linkage (UPGMA) algorithm in Mothur and an OTU cutoff was set to 0.1. The data were classified using the classify.seqs command in Mothur and the RDP training set 14. The default classify.seqs command uses the Wang et al. (2007) method to query the sequence 8-mer by 8-mer. The method calculates the probability a sequence from a given taxonomy contains a specific 8-mer and the probability a sequence belongs to a specific taxonomy based on its 8-mer set. In this manner, the query sequence was then assigned to the taxon with the highest probability. The cutoff for the highest probability was set to 90 percentile. Rarefaction and diversity indices were calculated using rarefaction.single and summary.single commands in Mothur.

### Amplicon Library Comparisons

Filtered 16S rRNA gene amplicon libraries were retrieved from the Sequence Read Archive (SRA) through NCBI for representation of the following environmental communities: Wastewater treatment plant (accession # PRJNA285699; Mayer et al., 2015), Mid-Atlantic Bight and Sargasso Sea communities (accession # PRJNA232722), Northern Mexico groundwaters (accession # PRJNA253631; Navarro-Noya et al., 2013). Coastal tidal marsh (Louisiana, United States) communities impacted by the Deepwater Horizon oil spill in 2010 (accession # PRJNA270760). Communities from a membrane bioreactor and associated treatment units during degradation of 1,2-dichloroethane (accession # PRJNA171127). Karst groundwater from the Edwards Aquifer in Texas, United States (accession # PRJNA82945). Terrestrial subsurface bacterial communities at a nuclear legacy waste site, at the Oak Ridge Integrated Field Research Challenge (ORIFRC) site in Oak Ridge, TN, United States (accession # PRJEB2767). Bacterial communities in a bio-reduced uranium contaminated site after re-oxidation (accession # PRJNA338649). Oligotrophic (Lake Brienz) and eutrophic (Lake Zug) lake habitats in Switzerland (accession # PRJNA188932). An acid mine draining pond located at the Copper Cliff South Mine, Copper Cliff, ON, Canada (accession # PRJNA212787), and the Red Sea Metagenomic Project (accession # PRJNA193416). Amplicon libraries were processed identically through the MG-RAST analysis pipeline (Meyer et al., 2008) to assess sequence quality, and to remove artificially replicated sequences, short sequences (multiplication of standard deviation for length cutoff of 2.0), and sequences with ambiguous bp (non-ACGT; maximum allowed number of ambiguous base pair was set to 5). The Greengenes and RDP databases were used as the annotation sources for the 16S rRNA genes, with minimum sequence identity of 97%, maximum e-value cutoff at 10–5, and minimum aligned sequence length of 100 bases. Only ribosomal gene sequences in the Domain Bacteria were retained (Eukaryote and Archaea were excluded). The abundance counts were log_2_ normalized (mean of zero and standard deviation of one) as previously described (Pozhitkov et al., 2015; Noble et al., 2016). Specifically, abundances were increased by one, log transformed, and centered to produce relative values. In order to standardize relative values, they were divided by the standard deviation of the log values.

### Principal Component and Cluster Analyses

Orthogonal transformation to their principal components (PC) and cluster analysis of the annotated genera abundances was conducted using normalized abundances. Principal component analysis (PCA) was determined using the matrix of Euclidean distances. The data were graphed on a two-dimensional ordination plot. Hierarchical clustering was conducted by using Average (UPGMA) and Wards linkage methods.

## Results and Discussion

**FIGURE 1:**
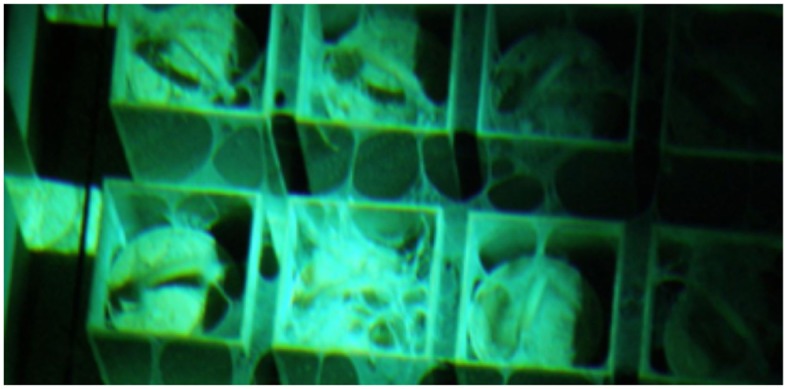
Underwater view of spent nuclear fuel canisters supported in a storage rack. Anomalous flocculent can be seen spanning the tops of individual fuel canisters and spreading between them along the storage rack.

Visual inspection of the SNF storage basin revealed that the precipitant occupied an estimated 7% of the total basin area, and the majority of the observed precipitant was physically associated with stored fuel components. A typical view of the flocculent associated with stored fuel is provided in **Figure 1**. No obvious correlations could be drawn to fuel inventories, age, or physical location in the storage basin. Complete sampling and thorough analysis of the entire SNF storage basin was not permitted because of safety, logistical or operational challenges associated with this facility.

Limited access for sampling SNF storage basins is an important issue. Water quality determinations for this system customarily rely upon a limited number of water samples, typically taken near the surface, for chemistry measurements, and metallurgical analysis of alloy coupons following short-term incubation in the water column (Santo Domingo et al., 1998). The inherent assumptions of this sampling scheme are that near surface waters are representative of the entire basin, and that suspended coupons are adequate analogs for the SNFs that are stored in the basin. While convenient, this strategy downplays the significance to which water chemistry can vary throughout the basin, by depth, and in proximity to metal surfaces (Kopečni et al., 1997; International Atomic Energy Agency [IAEA], 1998; Flemming et al., 2002). The source of this variability is in large part due to the non-uniform mixing of water in different areas of the basin and storage configurations, resulting in areas of stagnant water (e.g., Kain et al., 2000). For example, the use of storage racks in the facility greatly restricted water circulation (**Figure 1**). As such, it is logical to assume that within the basin, gradients in temperature, radiation dose and oxidant concentrations exist; as well as areas that differential or preferentially accumulate sludge on stored materials. Thus, it stands to reason that the microbiology, particularly microbial sources of corrosion, can also be highly variable and heterogeneous in these systems. All of these factors can contribute to the creation of localized areas having aggressive water chemistries and thus, conditions that are conducive to crevice or pitting corrosion of alloy cladding or other stored materials. Appropriate characterization and monitoring of water quality would require considerably more effort and expense to properly sample large complex basins; however, standard practices do not provide adequate representation of the aqueous environments where spent fuel and nuclear materials reside (Kain et al., 2000).

### Total Carbon Analysis

**FIGURE 2:**
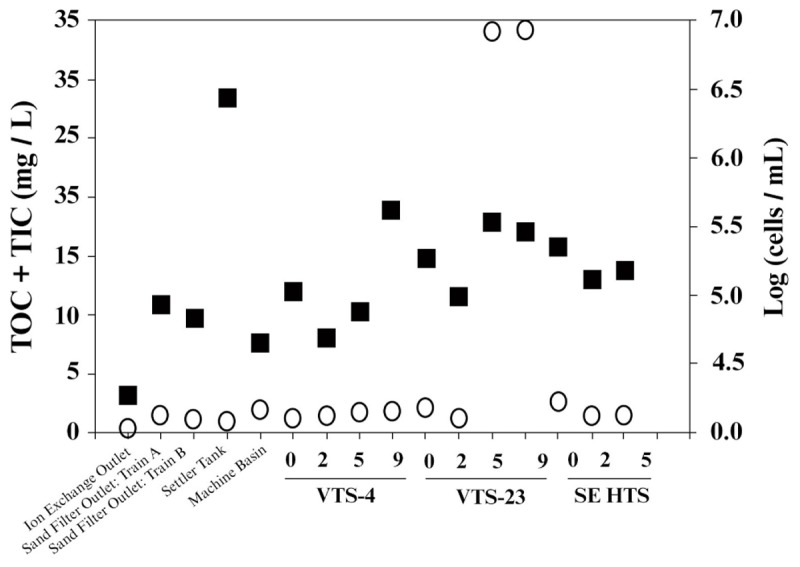
Carbon [total organic carbon (TOC) + total inorganic carbon (TIC); open circle, 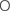] and bacterial cell density [log transformed (cells/mL); closed square, 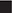] measured from basin water collected from operational/mechanical units that service the storage basin proper, as well as three distinct spent nuclear fuel storage locations (VTS-4, VTS-23, and SE HTS) at depth (meters).

Consistent with strict water quality management of the spent fuel storage basin, TOC measured from water samples taken throughout the entire facility were generally low. Averaged TOC was 4.2 mg/L (SD ± 10.6), which included samples from filtration systems and mechanical filtration units (**Figure 2**). TOC values within the storage basin where SNF and materials are kept averaged 6.08 ± 12.5 mg/L. Two sampling depths from a single location were outliers (discussed below), and if these values were removed the TOC for the storage basin averaged 0.45 ± 0.27 mg/L. This range of TOC values approaches the theoretical limit for microbial growth and energetics (Van der Kooij, 1992) and are analogous to values typical of low energy subsurface groundwater (Chapelle et al., 2009) often characterized by low bacterial activity and cell density. Numerous other SNF storage facilities reporting TOC values within this range, however, have demonstrated prolific diversity of planktonic and biofilm forming microbial communities (Masurat et al., 2005; Sarró et al., 2005; Tišáková et al., 2013). The notable exception to the measured low TOC trend, though, was a single SNF storage site, VTS-23, which had 31 mg/L TOC at 5.5 and 9 m depths. These high values for TOC were quite unexpected and are more typical of TOC levels from terrestrially impacted surface waters receiving photosynthetic inputs (Chapelle et al., 2009). TOC measured from collected surface water at site VTS-23 was considerably lower (0.88 mg/L), but still nearly twice as high as the low average TOC for all other spent fuel storage locations sampled within the basin (0.45 ± 0.27 mg/L). The highest measured TIC values were also taken at site VTS-23 at a sampling depth of 9 m (2.91 mg/L). Averaged TIC at all other spent fuel storage locations and depths within the basin was 1.16 ± 0.54 mg/L.

Very high carbon content was measured from the precipitant material harvested from the basin. TIC and TOC values from this material were 131 μg/g (wet weight) and 883 μg/g (weight wet), respectively. We conservatively assume that the localization of high levels of organic carbon in water samples taken from VTS-23 was most likely due to inadvertent collection of precipitant which was prevalent throughout the vertical tube storage (VTS) area in the basin.

### Total Cell Counts

Bacterial cell counts in the basin water varied considerably among the sampled locations throughout the facility (**Figure 2**); ranging from 4.3 cells/mL (log scale) [1.8 × 10^3^ cells/mL] to 6.5 cells/mL (log scale) [2.8 × 10^5^ cells/mL]. The lowest cell counts were recorded from effluent from the continuous deionizer; while the highest levels were measured from the back flush settler basin. Averaged cell numbers within the spent fuel storage locations of the basin proper were 5.2 ± 0.3 cells/mL (log scale) [1.9 ± 1.2 × 10^4^ cells/mL]. Suspended bacterial cell densities have been shown to fluctuate, though these estimates are within range of values reported previously for this system (Santo Domingo et al., 1998). Because bioavailable nutrients are generally limited in this system, we would expect organic carbon to effectively control bacterial growth and surface colonization in the basin. Total cell counts showed no significant correlation to total carbon or TOC for the sampled locations and depths included in this study (**Figure 2**). Conversely, an inverse relationship between cell densities and TIC was evident (*R*^2^ = 0.5). These results imply that additional environmental controls may be contributing to the measured biogeochemical phenomena occurring in the basin.

### Elemental and Mineralogical Composition of Precipitates

**FIGURE 3:**
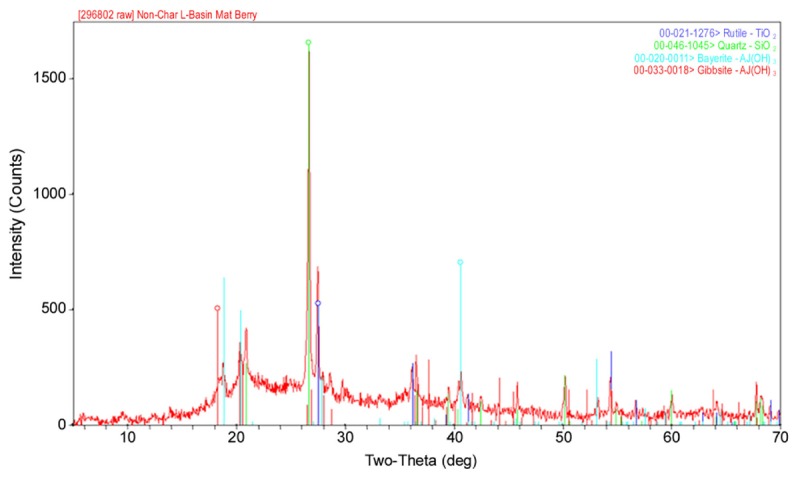
A representative XRD spectra of a composite sample of the precipitate collected from multiple locations throughout the spent nuclear fuel storage basin.

As shown in **Table 1**, the major elements detected from precipitant collected throughout the SNF storage basin included silicon, aluminum, titanium, and iron with lower amounts of zinc, sulfur, phosphorus, manganese, magnesium, potassium, calcium, and barium. Trace amounts of cobalt, chromium, copper, lanthanum, lithium, sodium, strontium, uranium, vanadium, and zirconium were also measured. The presence of silicon, titanium, and iron could have come from the sand bed filter in the basin, and aluminum most likely derived from the large quantity of aluminum materials present in the basin. The interior walls of the basin are coated in white paint that could have released titanium to the water. Iron, chromium, vanadium, zinc, and lanthanum are components of the stainless steel alloys that are used in a variety of materials as well as storage racks in the basin. Finally, barium and strontium likely existed as naturally occurring stable isotopes, but a small fraction likely originated as fission products from the uranium based nuclear fuels. We suspect that many of the elements detected are radioisotopes, though radiochemical analyses were not performed. XRD analysis was conducted on a composite precipitate sample collected throughout the basin. Multiple sampling locations were used to provide sufficient mass for the analysis and to provide a general measure of the dominant mineral precipitates in the basin. Major peaks were identified as rutile [TiO_2_], quartz [SiO_2_], bayerite [α-Al(OH)_3_] and gibbsite [Al_2_O_3_] (**Figure 3**).

**Table 1:** Elemental concentration (μg/g – w/wt) of precipitates collected throughout the spent nuclear storage basin.

Element	Concentration(μg/g – w/wt)	Element	Concentration (μg/g – w/wt)	Element	Concentration (μg/g – w/wt)	Element	Concentration (μg/g – w/wt)
Si	7960	P	267	Li	72.4	U	32.9
Al	6590	S	182	Na	45.1	Ni	12.7
Fe	4110	Mg	110	Cr	43.1	Sr	4.26
Ti	1060	Ba	99.9	Co	42.9	La	1.1
Ca	355	Mn	98.5	Pb	41.6	V	1.4
Zn	268	K	89.7	Cu	37.8		

*Only those elements above 1 μg/g – w/wt are reported. The elemental detection limits for these precipitates were <0.1% wt*.

Cladding material used for nuclear fuel rod construction is intended to physically contain fissile fuels and prevent the release of radioactive fission products from escaping into the reactor coolant or in this case, storage basin, during its production life in a reactor or throughout long term wet storage. Previous studies have shown that samples collected from a SNF storage basin or analyzed after short term incubations within the basin generally maintain compositional or elemental hallmarks that are consistent with exposure to irradiated nuclear fuels and materials (Sarró et al., 2003, 2005; Chicote et al., 2005; Tišáková et al., 2013). For example, ^235^U fission and neutron activation products were detected spectroscopically as relatively minor signals from the precipitant material that was sampled throughout the basin. The source of aluminum in the water is presumably the oxide film covering aluminum components being stored in the basin. Disruption of this protective layer could be attributed to numerous operational parameters, mechanical damage or chemical reactivity (e.g., Fe acting as a cathode); however, the exact source(s) remain unidentified (Louthan, 1997; Kain et al., 2000; Motta et al., 2015; Filburn and Bullard, 2016). A primary concern would be that bacterial colonization and excretion of metabolic reactants (e.g., oxidants, organic acids, reduced redox metals) could intensify chemical corrosion of fuels being stored in the basin over long periods of time prior to final disposition. It is clear, though, that aluminum levels in the water do become sufficiently high over time, leading to aluminum hydroxide polymerization and crystallization with age. We surmise that aluminum ions in the water have reacted to form the observed precipitate that appears to be colloidally dispersed, electrically attracted, and conditionally stabilized by physical-chemical conditions that we expect to be quite variable on a localized basis. Ionic composition of the water, organic ligands, pH, and temperature can all have a strong impact on aluminum polymerization; particularly, in the formation of mononuclear and polynuclear aluminum complexes [Al_n_(OH)_n_] that differ in solubility, though over time these reactions produce amorphous to crystalline phases (e.g., bayerite and gibbsite, respectively) (Hem and Roberson, 1967; Hem, 1968). Similar in process to chemical flocculation used in wastewater treatment and purification; we would predict that aluminum hydroxide agglomerations concentrate organic ligands, inorganic and organic colloids (including bacterial cells) from the water column by electrostatic forces and macromolecular coordination (Lartiges et al., 1997; Tripathy and De, 2006; Schneider et al., 2010). Viewed in this light, one could consider these episodic events of inorganic coagulation (i.e., precipitant formation) as a means of removing particulates and dissolved organics from the water, thus contributing to improved water quality.

### Analysis of the Storage Basin Microbial Community

General features of the amplicon pyrosequencing datasets generated from precipitant material collected throughout the SNF storage basin are shown in **Table 2**. The amplicon libraries contained 30,810 (59.5%) and 46,715 (95.2%) reads and 12,384 (40.2%) and 14,664 (13.4%) unique reads, respectively. Singletons made up 35.8% and 27.0% of the reads, respectively. Most of the sequences were classified into the Kingdom Bacteria (96.2 and 95.9%, respectively) and the remainder were classified as unknown.

**Table 2:** 16S rRNA gene amplicon pyrosequencing statistics.

Primers (16S rRNA region)	27F/338R (V1–V2)	520F/802R (V4–V5)
Reads	51,781	49,093
Total trimmed reads	30,810	46,715
Classified unique	12,384	14,664
Singletons	11,037	12,598
Doubletons	689	974
Bacteria	11,917	14,061
Unknown	467	602

Three bacterial diversity indices (i.e., Chao1, ACE, Shannon) were used to characterize the amplicon libraries (**Table 3**). The Chao1 and ACE estimates species richness while the Shannon index combines species richness and abundance into a single value of evenness (Shannon, 1948). The Chao1 differs from ACE because it estimates the total number of species present in a community based on the number of rare species (Chao, 1987), while ACE estimates the total number of species based on all species with fewer than 10 individuals (Chao and Lee, 1992). For the Shannon index, biological communities dominated by a few species have a low evenness, while those having species that are equally distributed exhibit a high evenness.

**Table 3:** Measures of alpha-diversity of samples characterized for three OTU definitions^a^.

Sample	No. of unique reads	0.01			0.02			0.03		
		OTU	Chao	Shannon	OTU	Chao	Shannon	OTU	Chao	Shannon
27F/338R	12,384	6,890	38,163	5.75	5,008	23,546	5.24	4,002	17,141	4.95
520F/802R	14,664	8,413	32,875	5.14	5,508	19,126	4.16	4,052	14,632	3.81

^a^*0.01, 0.02, and 0.03 are the OTU cutoffs in distance units, OTU signifies the number of OTUs observed, Chao signifies the Chao1 estimated minimum number of OTUs and Shannon signifies the Shannon diversity index*.

**FIGURE 4:**
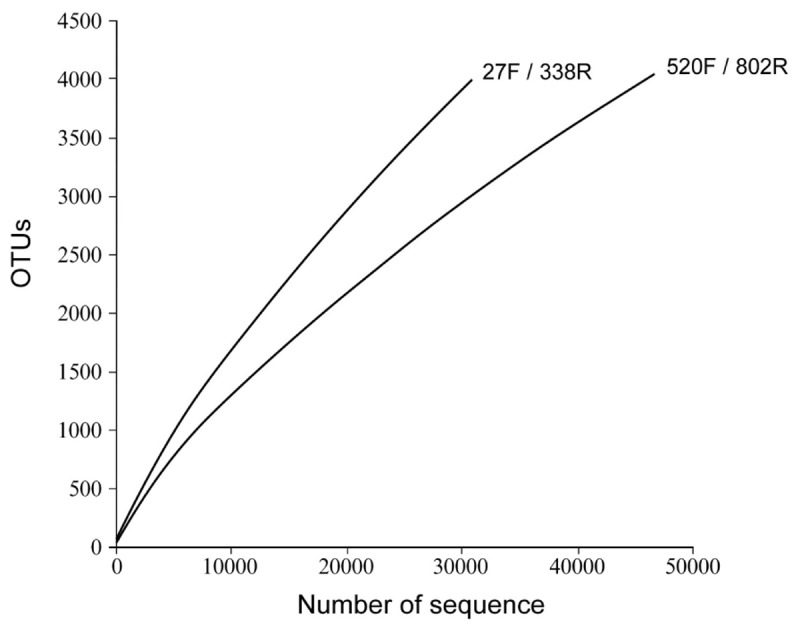
Rarefaction curves of OTU diversity defined at 0.03 distance cutoff for each of the 16S rRNA gene amplicon libraries prepared from flocculent material collected throughout the spent nuclear fuel storage basin.

The richness and diversity of the two libraries were calculated at OTU cutoffs of 0.01, 0.02, and 0.3 distance units using the number of observed OTUs, Chao1 estimated minimum number of OTUs, and a non-parametric Shannon diversity index (**Table 3**). Although library 27F/338R had more unique sequences than library 520F/802R, the number of OTUs changed with the cutoff thresholds, with library 27F/338R having fewer OTUs than library 520F/802R. Library 27F/338R consistently had higher alpha diversities than library 520F/802R. Rarefaction curves for a 0.03 distance cutoff show that since neither sample approached an asymptote, a greater sequencing effort is warranted (**Figure 4**).

Overall, analysis of the amplicon libraries revealed some obvious primer set specific biases. While the dominant phyla in both libraries were *Proteobacteria* and *Acidobacteria*; library 27F/338R represented significantly more sequences corresponding to Phylum *Nitrospirae* (12.8%) and *Actinobacteria* (2.5%) than library 520F/802R (0.5 and 0.3%, respectively). Importantly, the utilization of primer sets targeting multiple hypervariable regions of the 16S rRNA gene contributes a more complete description of microbial community composition than either primer set used alone.

Taxonomic binning results for quality amplicons generated from the precipitant collected throughout the SNF storage facility are summarized in **Table 4**. 16S rRNA gene sequences aligned most abundantly within the families *Burkholderiaceae* (23%), *Comamonadaceae* (6%), *Hyphomicrobiaceae* (17%), and *Nitrospiraceae* (23%). These four groupings accounted for 70% of all 16S rRNA gene amplicons when categorized to the family level. Sequences from two distinct genera were described, *Nitrospira* spp. (35%) and *Pedomicrobium* spp. (23%), comprising nearly 60% of all amplicons at the genus level. Overall, the physical–chemical and compositional descriptions of this SNF storage basin and its microbial community show specific consistencies with other published accounts (Sarró et al., 2003; Galès et al., 2004; Chicote et al., 2005). These consistencies reinforce the notion that a signature microbiological community exists for this unique, engineered environment; however, this is the first study applying amplicon sequencing to permit microbiome level comparisons to be made across a varied environmental landscape.

**Table 4:** Taxonomic binning of amplicons from precipitant sampled throughout the nuclear fuel storage basin.

Phylum	Family	Genera
Acidobacteria (10%)^a^	–	–
Gp3 (16%)	–	–
Gp6 (2%)	–	–
Gp2 (1%)	–	–
Gp1 (1%)	–	–
Planctomycetes (1%)	Planctomycetaceae (2%)	*Gemmata* (1%)
Actinobacteria (1%)	Mycobacteriaceae (1%)	*Mycobacterium* (2%)
Actinobacteria (2%)	–	–
Sphingobacteria (2%)	Chitinophagaceae (4%)	–
	Sphingomonadaceae (1%)	
Deinococcus–Thermus (2%)	Thermaceae (2%)	*Meiothermus* (2%)
Nitrospirae (12%)	Nitrospiraceae (23%)	*Nitrospira* (35%)
Proteobacteria (83%)	–	–
α (18%)	Hyphomicrobiaceae (17%)	*Pedomicrobium* (23%)
		*Hyphomicrobium* (1%)
	Methylobacteriaceae (3%)	*Methylobacterium* (2%)
	Bradyrhizobiaceae (9%)	*Bradyrhizobium* (4%)
β (22%)	Burkholderiales (27%)	*Burkholderia* (6%)
	Gallionellaceae (2%)	*Sideroxydans* (3%)
	Comamonadaceae (6%)	*Curvibacter* (4%)
		*Pelomonas* (4%)
		*Ideonella* (7%)
δ (1%)	–	–
γ (23%)	–	–

^a^*The percentage of total amplicons corresponding to each taxonomic assignment (OTU cutoff 0.1) are provided in parenthesis. Taxonomic bins containing <1% to total amplicons are not shown. ‘–’ denotes that no amplicons were confidently assigned*.

Sequences corresponding to the *Hyphomicrobiaceae* accounted for approximately 17% of all 16S rRNA sequences when categorized to the family level. Sequences from four distinct genera were described (*Aquabacterium* spp., *Hyphomicrobium* spp., *Pedomicrobium* spp., *Rhodoplanes* spp.) though *Pedomicrobium* spp. was the most abundant; comprising 23% of these sequences at the genus level. These alphaproteobacteria are characterized for a dimorphic lifecycle (hyphal – budding) and are prolific biofilm formers in many aquatic ecosystems, but they are a particular nuisance in engineered environments such as water distribution systems (Sly et al., 1988a, 1989) and hydroelectric pipes (Tyler and Marshall, 1967). *Pedomicrobium* spp. have been characterized for Mn(II) oxidation, and for extracellular polysaccharides binding metal (Mn, Fe) colloids. Based on strong correlations thus far identified in the published literature, an abundance of *Pedomicrobium* spp. in the system could be interpreted as a bio-signature that conditions are highly favorable for biofilm formation as a consequence of unchlorinated water and/or low water velocity (Sly et al., 1988b), or as a predictor of metal (Mn or Fe) oxide deposition onto metal surfaces or the basin floor (Gerbers, 1981; Sly et al., 1990). Both of these potential outcomes could have significant impacts on the deterioration of spent nuclear materials and storage systems (Ford and Mitchell, 1990; Gu, 2012).

Briefly, the *Nitrospira* spp. are the dominant nitrite oxidizing bacteria in a variety of aquatic habitats, as well as wastewater treatment systems and bioreactors (Hovanec et al., 1998). These microbes are chemolithoautotrophic, but they can grow mixotrophically on a variety of simple organic compounds in aerobic conditions. An abundance of recovered sequences corresponding to *Nitrospira* spp. immediately suggests that oxidative cycling of nitrogen is occurring in the basin; though we did not recover sequences corresponding to confirmed ammonia oxidizing bacteria (*Nitrosomonas* spp.; Archaea were not examined as part of this study) or companion nitrite oxidizers belonging to the *Nitrobacter* spp. While not measured in this study, we would anticipate that concentrations of NO_2_^-^ and NO_3_^-^ to be consistently low, along with other anions, on account of continuous operation of the deionizing system. As discussed in the recent review by Daims et al. (2016), there is growing recognition that nitrite-oxidizing bacteria are not as metabolically constrained as previously thought; thus, alternative lifestyles that are altogether uncoupled from NO_2_^-^ is a real possibility. For example, radiolysis of water produces a reliable supply of H_2_ in SNF storage basins which could be used as an energetically favorable metabolism for oxygen respiring nitrite-oxidizing bacteria (Marks et al., 2012). An explanation for the abundance of *Nitrospira* spp. sequences in this nuclear storage basin should be pursued. If analogies can be drawn from a recent study describing *Nitrospira* spp. dominance in an oligotrophic, thermal artisan spring (Marks et al., 2012); then, nitrifying bacteria may comprise important primary producers in this engineered ecosystem.

Sequences corresponding to *Burkholderia* spp. and *Bradyrhizobium* spp. comprised a relatively minor component of our amplicon libraries at the genus level but may be of particular interest as taxonomic signatures of bacteria inhabiting extreme low nutrient environments. Studies have reported on the reliable detection of these taxa in ultrapure water systems (Kulakov et al., 2002) and drinking water (Li et al., 2010; Navarro-Noya et al., 2013). Galès et al. (2004) isolated a *Burkholderia* sp. and *Ralstonia* sp. from an irradiated fuel storage pool by autotrophic enrichment, and demonstrated hydrogenase activity for both strains under oxic conditions. Microbes inhabiting engineered or geologic nuclear waste repositories are likely to be well adapted to an environment having high concentrations of hydrogen and oxygen with no significant inputs of organic carbon (Pedersen, 1993; Wang and Francis, 2005). Chemolithotrophy should be the foundational ecological metabolism for this and other oligotrophic, nuclear waste environments; and once established, conditions should permit the ecological and metabolic succession of mixotrophic and heterotrophic taxa. To this point, sequences corresponding to the *Gallionella* spp. and *Sideroxydans* spp. were also relatively minor components of the amplicon libraries but the abundance of these taxa have been shown to correlate with iron levels (Li et al., 2010; Navarro-Noya et al., 2013) which were high for this system. *Gallionella* spp. and *Sideroxydans* spp. are micro-aerobic iron oxidizers that can become prolific biofilm formers and have been shown to concentrate a variety of metals (e.g., Fe, lanthanides and actinides) along the length of its stalked cellular structures (Anderson and Pedersen, 2003). Detailed studies are needed to resolve the temporal development and maturation of a microbial community in this system but recognizing the scenario under which this basin was sampled (i.e., the proliferation of a heterogeneous precipitant throughout) the existence of a taxonomically, and presumably metabolically, diverse community was evident.

The SNF storage basin libraries were compared to reference datasets composed of amplicon libraries derived from marine waters (33 libraries), wastewater (35 libraries), groundwater (45 libraries), and surface waters (16 libraries). A 2-dimensional PCA plot for all 16S rRNA amplicon libraries is shown in Supplementary Figure 1. PCA yielded similar results when the 16S rRNA amplicon libraries were annotated using Greengenes (PC1 = 35%, PC2 = 4%) or RDP (PC1 = 33%, PC2 = 4%). Two PC axes could only explain 39–37% of the total variability in the data, meaning that the majority of the amplicon libraries could not be confidently resolved within a 2-dimensitional analysis space. The statistical power of PCA could almost certainly be improved by decreasing the size or content of our reference library but the conclusion drawn from these results clearly show that the specific variability (taxonomic diversity) between libraries was not significantly conserved (i.e., too high) to permit ordered separation of samples beyond a single PC axis.

**FIGURE 5:**
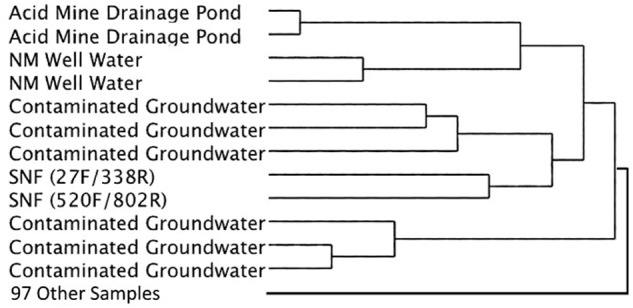
Simplified dendrogram showing hierarchical cluster analysis by the Ward’s minimum variance method using RDP taxonomic assignments.

Cluster analysis of the same datasets displayed as dendrograms, conversely, revealed that the SNF storage basin libraries (labeled K7 and K8) were distinctly different from the other samples in the reference library (**Figure 5**) regardless of annotation source (Greengenes or RDP) or linkage methods (UPGMA or Wards) employed (Supplementary Figures 2, 3). At a course level, though, the SNF storage basin libraries aggregated most closely with amplicon libraries produced from drinking water wells in Northern Mexico (accession PRJNA253631; Navarro-Noya et al., 2013), a karst aquifer system in Texas, United States (accession PRJNA82945) and a membrane bioreactor community employed to degrade organic contaminants (accession PRJNA171127). To determine the source of these differences, we compared amplicon abundance at the genus level for the SNF storage basin libraries compared to those of all reference datasets. We identified 27 genera in the SNF storage basin libraries that had maximum abundances and were distinct (i.e., bio-signatures). Interestingly, three of these genera were found to be numerically abundant amplicons from the SNF storage basin; these included *Methylobacterium* spp. (3.9%), *Nitrospira* spp. (35%), and *Meiothermus* spp. (2.4%). All remaining amplicons that differentiated the SNF basin library from other reference environments were categorized into low abundance genera. Several of these sequences could not be taxonomically assigned (i.e., unknowns) or fell into candidate genera; while the balance of sequences were taxonomically assigned to soil microbiota (*Streptosporangium* spp., *Solibacter* spp., *Myxobacteria* spp.) or commensals associated with plants (*Kitasatospora* spp., *Gluconacetobacter* spp.), animals and humans (*Ruminococcus* spp., *Megasphaera* spp., *Taylorella* spp., *Lamprocystis* spp.). These taxa represent unlikely signatures for this engineered environment, and were most likely introduced into the system by dust or debris contamination from air or plant workers, or perhaps as contamination from the packaging and shipment of nuclear materials prior to storage in the cooling basin.

While the available literature on the microbiology of SNF storage facilities are limited; it is certainly clear that the high purity water that is exposed to spent nuclear materials is not devoid of microbial inhabitants. In fact, a generalized description of the community, chiefly its primary constituents, is beginning to emerge. This realization should inform the expansion of future research into these systems by considering the characterization and sources of carbon and energy as one possible management strategy to restrict microbial growth and development in the SNF storage basin. First of note, there are numerous growth dependent studies (Sarró et al., 2003, 2007; Galès et al., 2004; Chicote et al., 2005) which have described the isolation of *Firmicutes*, chiefly bacilli, which would presumably have a clear competitive advantage in oligotrophic waters and against hazardous exposure to radiation and radionuclides. However, we would predict that spore forming microorganisms, while present, may be less important among the active microbial community in SNF storage facilities. The more comprehensive, and growth independent, description of the microbial community provided here by the application of amplicon sequencing supports this prediction as surprisingly few known spore forming bacteria were described. While this conclusion could be credited to methodological bias, it is certainly conceivable that environmental conditions are conducive to vegetative cell growth and activity in the basin water, or specifically in association with the microhabitat created by flocculated aluminum hydroxides.

The emerging consistencies in microbial community taxa reinforces the notion that microbiological community signatures do exists for this unique, engineered environment. Most notably, *Burkholderia/Ralstonia*, methylobacteria, sphingomonads, as well as *Actinobacteria* are all reoccurring taxa that are strongly associated with SNF storage basins. Importantly, many of these taxa have also been shown to form radionuclide accumulating biofilms on stainless steel surfaces during experimentation in which materials were suspended in the water of spent fuel storage basins (Sarró et al., 2003, 2005, 2007). The significance of this trait would need to be assessed against empirical data for actual spent nuclear materials, but the conservation of this phenotype among basin microbes would become increasingly concerning as materials age in wet storage over long periods of time thereby increasing the likelihood of biofilm formation and microbiologically influenced corrosion. Less commonly observed in published studies (cultivation dependent) but numerically abundant taxa in this study were the *Nitrospira* spp. and *Hyphomicrobium* spp. which are loosely associated with ‘dirty water.’ These taxa could signify secondary colonizers that respond to changing chemistry or declining water quality such may be the case here when aluminum hydroxide polymerization becomes widespread throughout the storage basin. As such, these microbiological signatures may be a symptom of having aluminum alloy clad fuels stored in this basin because, as state prior, these materials are not compatible with the most commonly used disinfectants for biological control of these facilities.

## Conclusion

This investigation provides the first description of the microbial community within a SNF storage facility using cultivation independent, next generation amplicon sequencing to reveal unexpected diversity within this extreme aqueous environment. The obvious purpose of this study was to investigate the observed proliferation of an amorphous white precipitant (aluminum hydroxides) throughout the storage basin, but through the course of this investigation, in part due to the application of new combinations of analytical tools, we gained an improved perspective of this engineered ecosystem. This study (even with relatively limited sampling) demonstrates that SNF storage basins are complex, dynamic systems, in which chemical, biological, and physical conditions vary spatially and evolve temporally. This revised perspective should have important implications for how water quality is currently managed and corrosion surveillance programs conducted in these facilities. Accordingly, the future opportunity afforded by this study employs next generation sequencing and computational analytics to advance biological monitoring through the development of reliable bio-signatures. Tractable signatures could be used in combination with traditional measurements for more intensive sampling and real-time monitoring to better anticipate changes to the condition of the water, or emergent properties therein, on relevant scales for the safe management of nuclear materials during wet storage. Appropriate monitoring of interim storage conditions of SNF basins is of increasing concern as global inventories increase and decisions for permanent stabilization and disposal continue to be delayed.

## Author Contributions

CB, PN, CM, DL, and DK contributed intellectual input and assistance to this study and manuscript preparation. CB developed the original framework. DL and DK contributed reagents and data analysis. PN performed statistical analysis and data integration. CB wrote the paper.

## Conflict of Interest Statement

The authors declare that the research was conducted in the absence of any commercial or financial relationships that could be construed as a potential conflict of interest.
