# Supplementary figures and images for: Amplicon Sequencing Reveals Microbiological Signatures in Spent Nuclear Fuel Storage Basins
Source: Front Microbiol. 2018 Mar 9;9:377. doi: 10.3389/fmicb.2018.00377 (PMC5854691; doi:10.3389/fmicb.2018.00377)

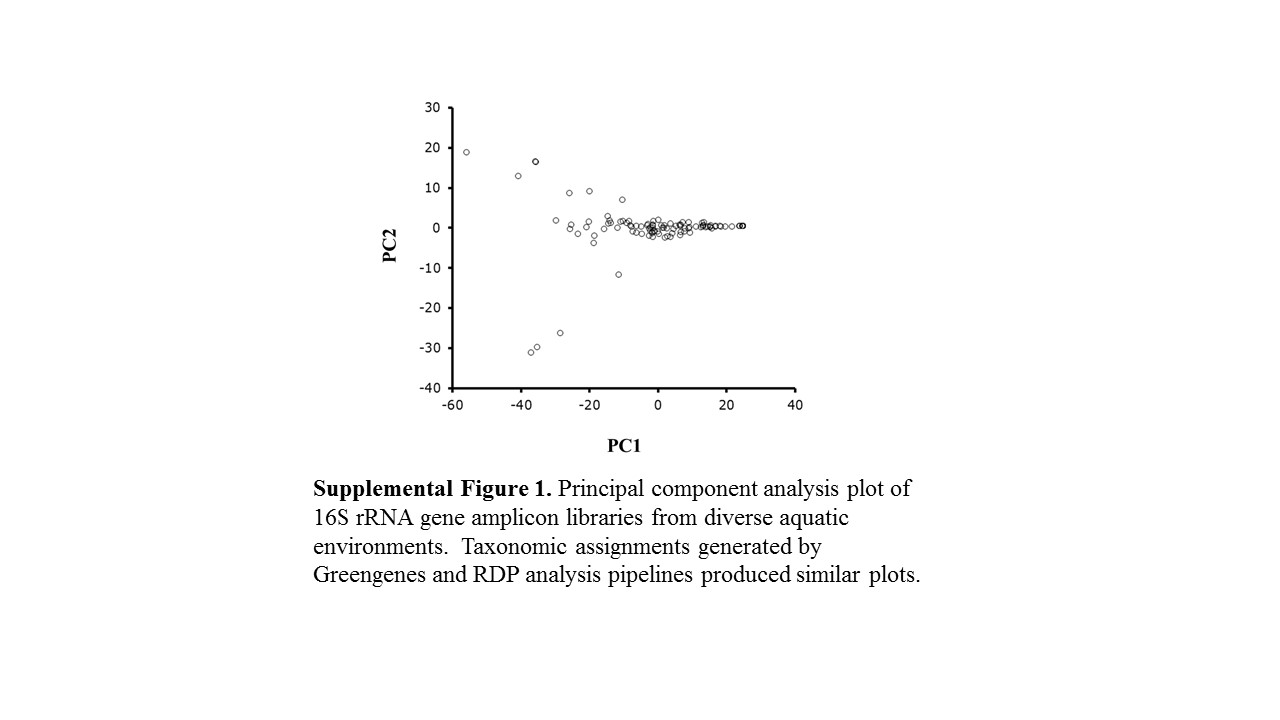

Supplement: Supplementary file 1 [file Image_1.JPEG]

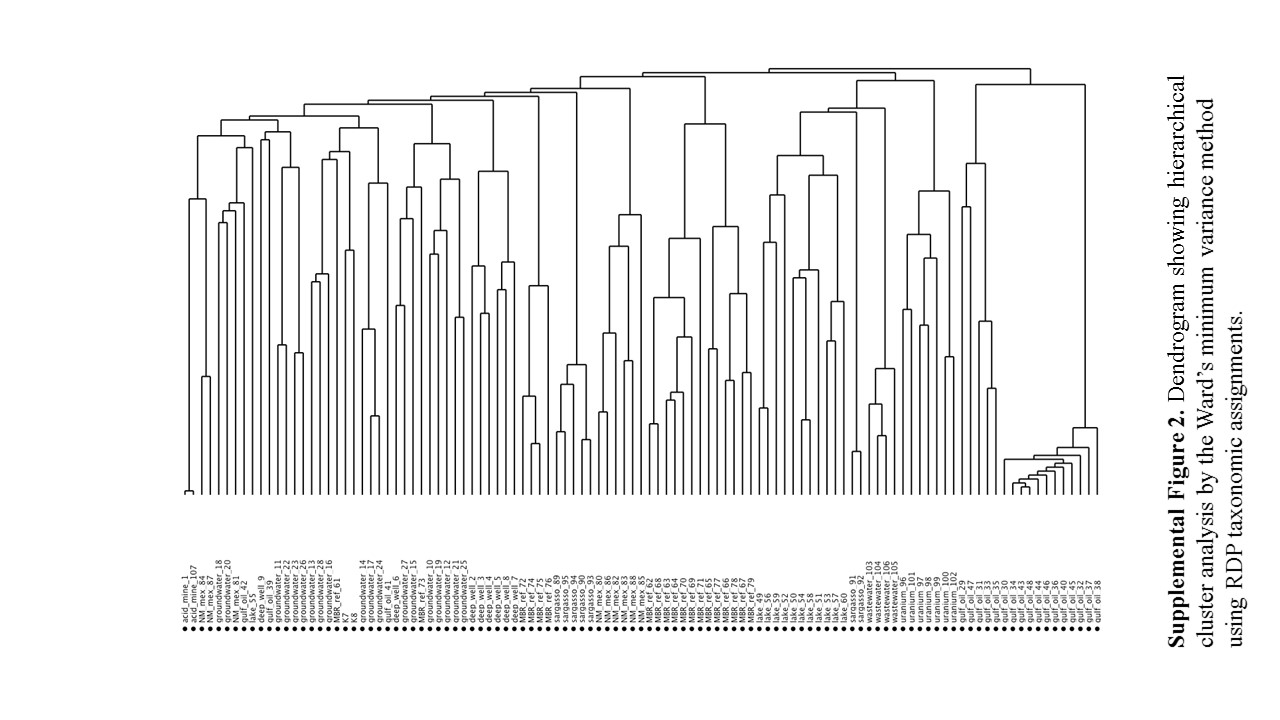

Supplement: Supplementary file 2 [file Image_2.JPEG]

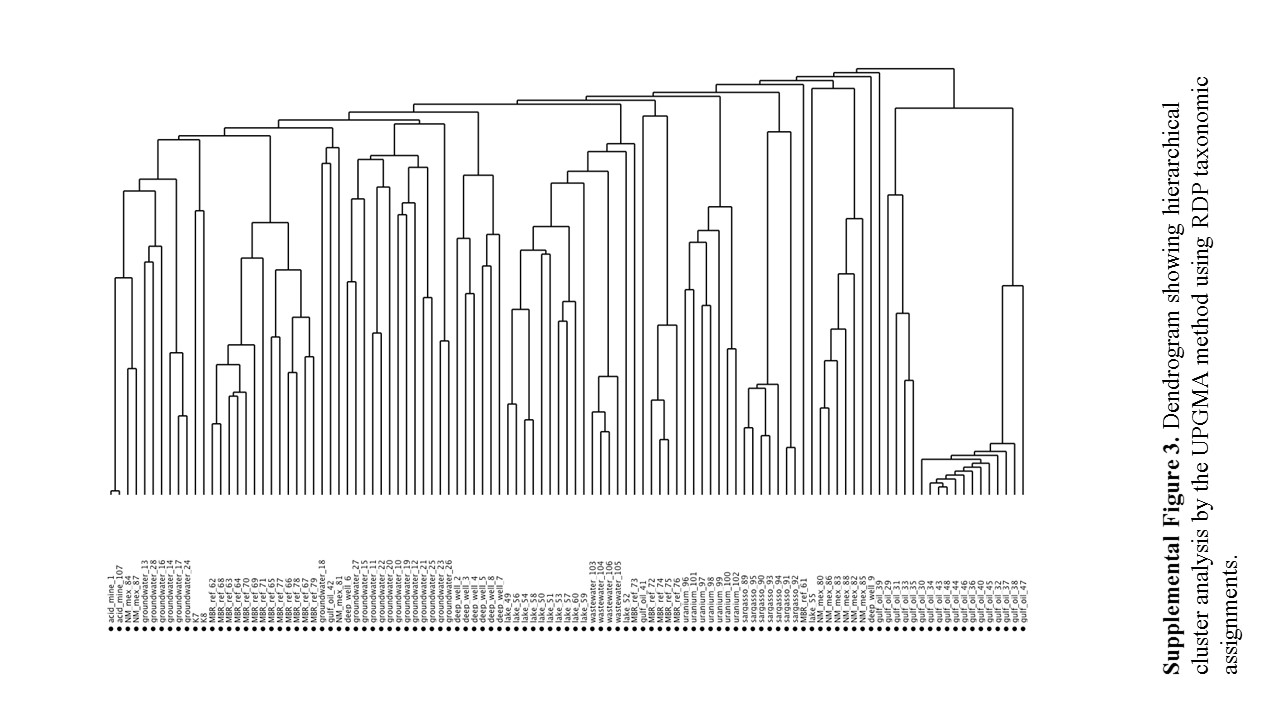

Supplement: Supplementary file 3 [file Image_3.JPEG]
